# Proton Pump Inhibitors and Osteoporosis: Is Collagen a Direct Target?

**DOI:** 10.3389/fendo.2020.00473

**Published:** 2020-07-22

**Authors:** Yohannes T. Ghebre

**Affiliations:** ^1^Department of Radiation Oncology, Baylor College of Medicine, Houston, TX, United States; ^2^Department of Medicine, Section on Pulmonary and Critical Care Medicine, Baylor College of Medicine, Houston, TX, United States

**Keywords:** proton pump inhibitors, osteoporosis, osteoporotic fractures, bone density, collagen

## Background

Proton pump inhibitors (PPIs) are FDA-approved drugs for the treatment of several gastric acid-related disorders including indigestion, peptic ulcers, Barrett's esophagus, Zollinger Ellison syndrome and *Helicobacter pylori* infection-induced gastritis (1). Accordingly, PPIs are among the most consumed drugs worldwide with an ever-increasing use from time to time (2). Currently, the global sales of prescription and over-the-counter PPIs is estimated to be about $14 billion dollars with ~113 million PPI prescriptions worldwide (3). One of the growing concerns with widespread PPI use is their inappropriate use for prolonged period of time to treat gastric acidity, as well as for indications that are not quite clear. By extension, one of the most significant medical concerns is the potential association of PPI use with increased risk for osteoporotic fractures including hip and vertebral fractures (4–6). Recently, a large meta-analysis study reported that the use of PPIs is significantly associated with increased risk of hip fracture (7). Accordingly, the US FDA has issued a safety warning label in 2010 to emphasize the increased risk of spine, wrist and hip fractures with PPI use. These concerns for musculoskeletal harm are almost exclusively believed to be due to reduced absorption of calcium presumably because PPIs cause low level of stomach acid (hypochlorhydria) that is believed to impair calcium solubility and cause malabsorption to exacerbate loss of bone mineral density secondary to low plasma calcium-induced hyperparathyroidism, osteoclast activation and bone resorption (8, 9). Intriguingly, there appears to be a trend toward increased bone fracture incidence with increased dose, adherence and duration of PPI use, but not the use of histamine H_2_-receptor antagonists (H2RAs; alternate antacids with similar acid-reducing capabilities as PPIs) (10), and that this risk is independent of changes in bone mineral density (4, 8, 11, 12) suggesting that the depletion of bone minerals such as calcium and vitamin D or suppression of gastric acidity is unlikely to be the driving mechanism by which PPIs increase the risk of osteoporotic fractures. Additional clinical studies have observed that PPI users are more likely to sustain fracture and start medications for osteoporosis than non-users (13), and that there was no fracture risk reduction with the use of hormone replacement therapy (HRT) if PPIs are concomitantly used (14) indicating the increased risk of fracture with PPI use and possible negation of the bone protective effect of HRT. Alternative explanations for the increased risk of osteoporotic fracture with PPI use include inhibition of osteoblast or osteoclast activity to dysregulate bone remodeling (15, 16), and increased rate of bone resorption (17). However, clinical cohorts comparing long term PPI users with non-users found no difference in the rate of bone density loss including in perimenopause women who are at increased risk of bone loss and potentially susceptible to an adverse effect (18, 19). Intriguingly, Jo et al. (17) reported that the use of PPI was associated with increased release of calcium and deoxypyridinoline; a crosslink that provides structural stiffness to the type I collagen found in bones, suggesting that major components of the bone might be targeted by PPIs.

## Perspective

Recently, our cell biological and preclinical animal studies revealed that PPIs target major extracellular matrix (ECM) components such as collagen (20). Our *in vitro* studies in primary cells derived from human lungs indicate that PPIs inhibit the gene expression of collagen (e.g., collagen 1A1) and fibronectin, and PPI-treated lung fibroblasts show significantly reduced degree of proliferation and decreased collagen release (20). Our *in vivo* studies in an animal models exposed to chemotherapeutic drug (bleomycin) or radiation; stimuli known to induce fibrosis (ECM deposition), also showed that administration of the PPI esomeprazole significantly reduced collagen accumulation in the lungs and skin (20, 21). Other studies also reported that PPIs reduce collagen accumulation in the liver (22). Intriguingly, there is also a report that lung fibrosis patients who happened to be on PPIs had reduced fibrosis score on high resolution computed tomography (HRCT) scans (23) suggesting that PPIs might target collagen. Previously, Pullamsetti et al. (24) have reported that the expression of dimethylarginine dimethylaminohydrolase (DDAH), a cytoplasmic enzyme that is closely associated with inducible nitric oxide synthase (iNOS) to promote inflammatory and fibrotic phenotypes, is pathologically upregulated in the lungs of pulmonary fibrosis patients, and our biochemical and immunohistochemical studies (20, 21, 25) show that PPIs inhibit DDAH to reduce soluble and total collagen levels. Our recent studies in human lung cells also show that PPIs inhibit the gene expression of collagen types 1, 3, and 5. Accessible data from The Library of Integrative Network-based Cellular Signatures (LINCS) database (http://www.lincscloud.org) on lung cells treated with esomeprazole show that the PPI inhibits the expression of several ECM components including members of the collagen family such as collagen 3A1 (COL3A1), collagen 4A1 (COL4A1), collagen 5A1 (COL5A1), collagen 5A2 (COL5A2), and other myofibroblast markers (20, 26). Taken together, it appears that PPIs may actually target the ECM in general and members of the collagen family in particular to influence bone pathophysiology including increasing the risk of osteoporosis and osteoporotic fractures in patients. In the face of lack of plausible biological mechanism and conclusive evidence for causal relationship between PPI use and osteoporosis, the new mechanistic insights provided here need to be investigated further. In addition, the lack of positive correlation between bone mineral density and osteoporotic fracture in PPI users as described above invites a fresh perspective into looking at these two conditions independent of each other. Prospective studies should also be designed to isolate bone cells and evaluate the gene expression of members of the collagen family that constitute the ECM including collagen types 1, 3, and 5 in susceptible patients prior to, during, and after the completion of PPI use. Such clinical studies may be preceded by preclinical assays of primary human osteoblasts (27) cultured in the absence or presence of PPIs, and analyzed for the expression of ECM proteins. It is anticipated that such prospective studies will address important questions including whether PPIs have direct effect on collagen and/or other major ECM components.

## Conclusion

Since we cannot rule out the possibility of causal relationship between PPI use and osteoporosis, clinicians should be judicious with the prescription of PPIs and should vigilantly monitor at risk patients including older patients as well as long term PPI users.

## Author Contributions

The author confirms being the sole contributor of this work and has approved it for publication.

## Conflict of Interest

YG is an inventor on patents, owned by Stanford University and Baylor College of Medicine, that protect the use of agents, including proton pump inhibitors (PPIs), for therapeutic use of new indications.
